# Characterization of Bitumen Micro-Mechanical Behaviors Using AFM, Phase Dynamics Theory and MD Simulation

**DOI:** 10.3390/ma10020208

**Published:** 2017-02-21

**Authors:** Yue Hou, Linbing Wang, Dawei Wang, Meng Guo, Pengfei Liu, Jianxin Yu

**Affiliations:** 1 National Center for Materials Service Safety, University of Science and Technology Beijing, Beijing 100083, China; alladin@outlook.com (Y.H.); mguo@ustb.edu.cn (M.G.); 2 Joint USTB-Virginia Tech Lab on Multifunctional Materials, USTB, Beijing, Virginia Tech, Blacksburg, VA 24061, USA; wangl@vt.edu; 3 Institute of Highway Engineering, RWTH Aachen University, Mies-van-der-Rohe-Street 1, D52074 Aachen, Germany; liu@isac.rwth-aachen.de; 4 Center of Analysis and Measurement, Harbin Institute of Technology, Harbin 150001, China; yujx@hit.edu.cn

**Keywords:** Atomic Force Microscopy, bitumen, micro-mechanical behaviors, Phase Dynamics Theory, MD simulation

## Abstract

Fundamental understanding of micro-mechanical behaviors in bitumen, including phase separation, micro-friction, micro-abrasion, etc., can help the pavement engineers better understand the bitumen mechanical performances at macroscale. Recent researches show that the microstructure evolution in bitumen will directly affect its surface structure and micro-mechanical performance. In this study, the bitumen microstructure and micro-mechanical behaviors are studied using Atomic Force Microscopy (AFM) experiments, Phase Dynamics Theory and Molecular Dynamics (MD) Simulation. The AFM experiment results show that different phase-structure will occur at the surface of the bitumen samples under certain thermodynamic conditions at microscale. The phenomenon can be explained using the phase dynamics theory, where the effects of stability parameter and temperature on bitumen microstructure and micro-mechanical behavior are studied combined with MD Simulation. Simulation results show that the saturates phase, in contrast to the naphthene aromatics phase, plays a major role in bitumen micro-mechanical behavior. A high stress zone occurs at the interface between the saturates phase and the naphthene aromatics phase, which may form discontinuities that further affect the bitumen frictional performance.

## 1. Background and Introduction

Fundamental understanding of micro-mechanical behaviors in bitumen, including phase separation, micro-friction, micro-abrasion, etc., can help the pavement engineers better understand the bitumen mechanical performances at macroscale. There have been many researches in the bitumen and bituminous mixture mechanical performance evaluation at macro- and micro-scale. Bazlamit et al. studied the changes in bitumen pavement friction using laboratory experiments [1]. Fischer et al. evaluated the relationship between chemical compositions and bitumen micro-mechanical performance by Scanning Near-Field Optical Microscopy [2]. Al-Rub et al. proposed a micro-damage healing model which gives a more accurate fatigue life prediction in bituminous mixture [3]. Kanafi et al. studied the macro- and micro-texture evolution of the road pavement and the correlation with friction [4]. Their results show that the micro-mechanical behaviors of bitumen and bituminous mixture may affect the macro-mechanical performances. 

Meanwhile, researchers found that the chemical composition and microstructure of bitumen will significantly affect the micro-mechanical performance, including micro-friction behaviors, using advanced testing techniques. Bitumen is a complex mixture of hydrocarbons. Due to the advancements in microscopy technology, a series of experiment apparatuses including Atomic Force Microscopy (AFM) are developed and allow the researchers to analyze the microstructure of bitumen at a much smaller scale. AFM is capable to provide a topographic profile of the surface and to achieve a resolution of the bitumen surface structure down to a few nanometers. 

Loeber et al. observed the microstructure of bitumen referred as “Bee Structure” using scanning SEM (Scanning Electron Microscope) and AFM, and found the bitumen surface structure can be visualized by AFM [5]. Pauli et al. [6] and Jager et al. [7] found the same “Bee-Structure” of bitumen as Loeber confirmed that the “Bee Structures” were asphaltenes under AFM. Masson suggested that various bitumen domains can attribute to specific bitumen fractions [8]. Allen et al. investigated the microstructural composition of bitumen and the response of microstructural phases under load using AFM. They found that long-term aging evidently induces microstructural changes in bitumen and distinct properties of each phase [9]. McCarron et al. concluded that temperature has an effect on size and shape of bee structures of bitumen and confirmed that wax could indeed be responsible for bee structure formation [10]. Masson et al. [11], De Mores et al. [12] and Pauli et al. [13] utilized AFM to investigate the surface microstructure of bitumen. Dourado et al. conducted Finite Element simulations and AFM experiments and found that applying strain resulted in damage/phase concentrated in the interstitial zone between neighboring bee structures [14]. They also suggested that the evaluation of bitumen microstructure and micro-rheology is critical for understanding the mechanisms of damage evolution in bitumen [14]. 

According to the above literature review, even though much effort has been devoted, the relationship between the micro-mechanical behavior and microstructure/chemistry of bitumen is very complicated, still not clear, and under debate among researchers. Moreover, although the authors have previously analyzed the bitumen microstructure evolution using phase field method [15,16], the researches do not relate the microstructure to the frictional behavior and the determination of bitumen parameters in phase dynamics model is not given. To better understand the micro-mechanical behaviors in bitumen, in this study, the bitumen microstructure and micro-mechanical behaviors at the surface are studied using AFM experiments. Phase Dynamics Theory is employed to simulate the bitumen microstructure evolution. In Phase Dynamics Theory, a phase-field variable is used to identify different phases, based on which a free energy functional is constructed. The whole system evolves toward the direction which minimizes this free energy. To reasonably determine the parameters in the Phase Dynamics model, MD simulation is conducted based on the bitumen three-component molecular structure. Note that we are currently only dealing with the relationship between microstructure evolution and micro-mechanical behavior, and future studies should consider the contact effects [17,18,19]. The ultimate goal is to understand the effects of such microstructure on micro- and even macro-mechanical behaviors of bitumen and bituminous mixture. 

## 2. AFM Experiments

The AFM experiments are first conducted to characterize the microstructure of bitumen samples at microscale.

### 2.1. The Preparation of AFM Samples

**Figure 1:**
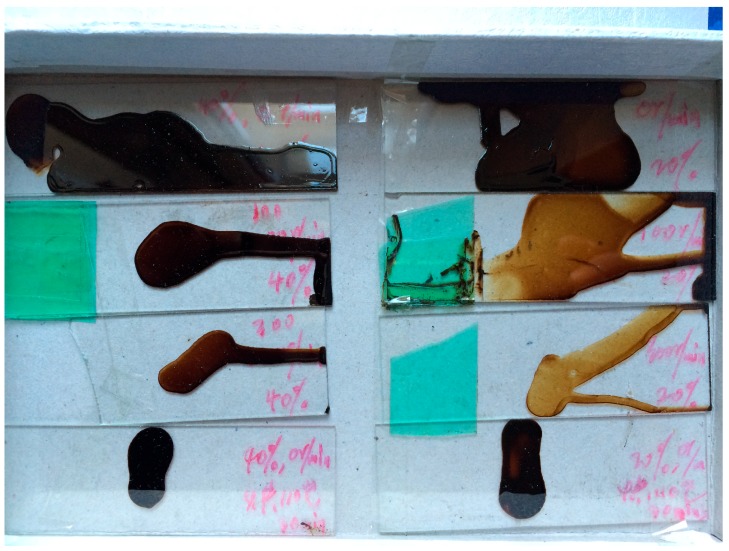
Preparation of bitumen samples.

The AFM samples are prepared using bitumen with penetration grade 90 as shown in Figure 1. Toluene was used to dissolve the bitumen in a magnetic stirring apparatus. The bitumen-toluene liquid with 20% concentration (mass of bitumen: mass of toluene = 1:4 was obtained after stirring 24 h in an airtight conical flask. The plastic head dropper was used to transfer a drop of bitumen-toluene liquid from the conical flask to the glass slice. Leave the glass slice with a drop of sample in the constant temperature drying oven to avoid dust pollution. The final tested sample was obtained after the toluene was evaporated completely (about seven days), leaving a film of bitumen adhered onto the glass slice. The bitumen ageing is not considered during these processes for simplicity, which should be given attention in future studies.

### 2.2. Selection of Test Parameters

**Figure 2:**
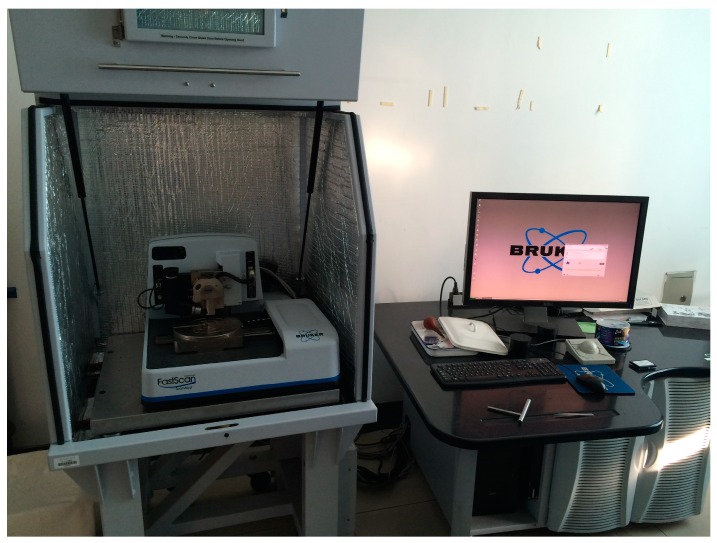
AFM instrument.

**Figure 3:**
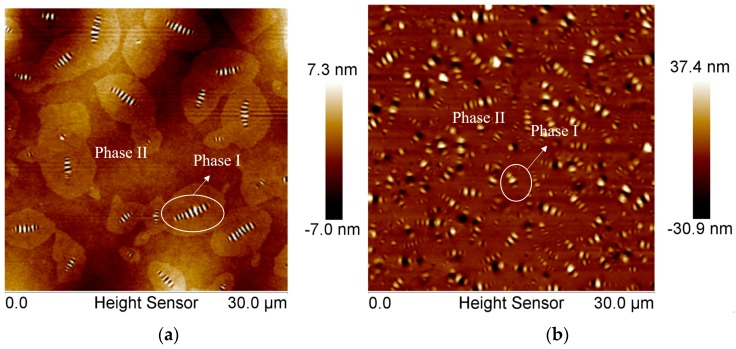
The nano-morphology of bitumen tested by AFM: (**a**) Type α; and (**b**) Type β.

Tapping mode was selected to measure the nano-morphology of bitumen in the tests. The cantilever end of AMF was RFESPA developed by Bruker, as shown in Figure 2 and Figure 3. Its nominal resonant frequency was 75 Hz, nominal elastic constant was 3 N/m (the obtained real elastic constant was 4.3 N/m by using Thermal-tune method). The Tip material was silicon, whose height was 15–20 µm, and nominal tip radius was 8 nm. 

### 2.3. AFM Results

The typical characterized morphology results are shown in Figure 3. In Figure 3, there are two types of bitumen morphology, Type α, and Type β. All the three AFM figures show that, under certain thermodynamic conditions, a complex microstructure evolution will occur in the bitumen sample, whereas there are different phases occurring in the sample, indicating the separation or uneven matter re-arrangements at microscale. Note that it is a bi-phase system that we are observing (phase I and phase II). Phase I is the bee-structure and Phase II is the background matrix. Although it is difficult to determine the exact chemical composition and micro-property of each phase, there have been significant progresses in this area [20,21].

**Figure 4:**
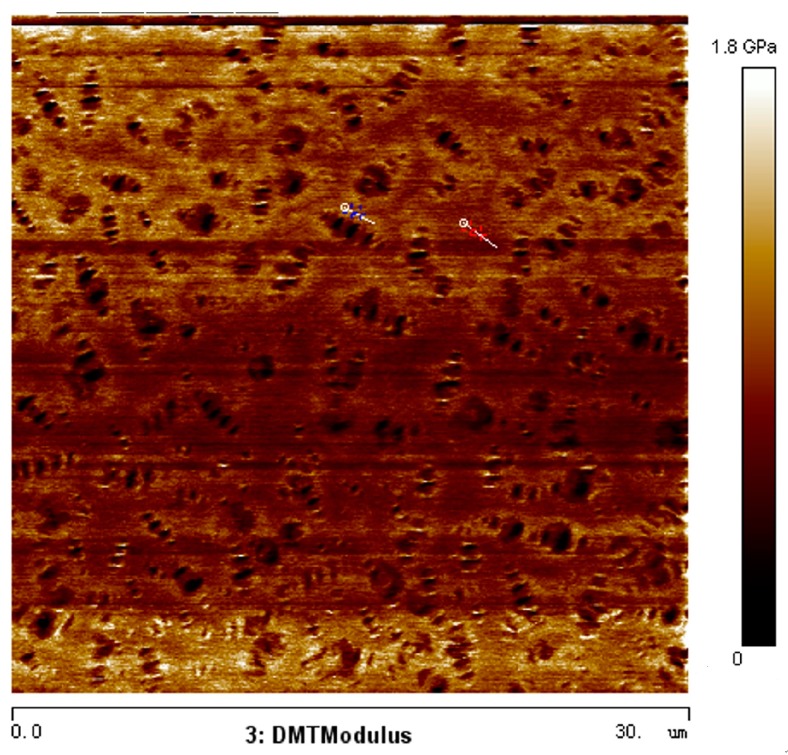
DMT modulus of bitumen characterized by AFM.

Figure 4 shows the Derjaguin-Muller-Toporov (DMT) modulus of bitumen samples at microscale. To obtain a material’s elastic modulus, the retraction curve can be fitted using different contact mechanics models, and DMT model is best for stiff materials with low adhesion. It is observed that the characterized modulus of bitumen in some regions is very large compared with the commonly used bitumen modulus at macroscale, which is mainly due to the size effect. It is easy to conclude that such uneven microstructure distribution of bitumen samples will definitely affect the bitumen micromechanical properties and even macro-mechanical properties, whereas such morphologies cannot be characterized by normal mathematical approaches. In this way, Phase Dynamics Theory and Molecular Dynamics (MD) Simulation are employed to investigate and simulate such microstructure phenomena. The Phase Dynamics Theory was first suggested by Cahn and Hilliard [22]. In this theory, a phase-field variable is used to identify different phases, based on which a free energy functional is constructed. The whole system evolves toward the direction which minimizes this free energy. To reasonably determine the parameters in the Phase Dynamics model, MD simulation is conducted based on the bitumen three-component molecular structure.

## 3. Phase Dynamics

It is discovered that different phases distribute on the bitumen samples surface in the AFM experiments, as shown in Figure 3, and such phenomenon will obviously affect the bitumen micromechanical behaviors [23,24,25]. Note that AFM only measures the surface properties, which is not the bulk properties. One possibility must be excluded before we begin our analysis. That is the phase separation of the “bumble-bees” structure may be caused by surface tension effect but not a “bulk” effect. Schmets et al. conducted Small Angle Neutron Scattering (SANS) experiments by comparing the SANS response with surface and bulk models and proved that the phase-separation features observed on the bitumen sample’s surface are also presenting in the bulk [23]. Therefore, the surface tension effect is not discussed in this study.

### 3.1. Bitumen Chemistry and Free Energy

Bitumen is one of the products of the petroleum industry and thus the components of bitumen are very complex. The oldest and the most common determination methods of bitumen composition is bitumen three-composition chemistry. In this method, bitumen is decomposed into three main groups of molecules: resin, oil and asphaltene. After that, Koots [26], Lian [27], and Hubbard [28] also contributed in this area. Some national standards also pay attention to this area. For instance, the Standard test methods of bitumen and bituminous mixture for Highway engineering in China can be used to determine the bitumen composition [29]. A summary of the three-composition of bitumen chemistry is shown in Table 1. Note that the bitumen chemistry can also be divided into two groups via solution in n-heptane: asphaltenes and maltenes. The maltenes are then divided into three groups of molecules: resins, aromatics, and saturates. 

Another approach to investigate the bitumen chemistry is to characterize bitumen chemically by its hydrocarbon class composition, e.g., three main constituent species including asphaltene, naphthene aromatics and saturates based on the asphalt molecular structure [30]. Different chemical components and the functional units (O, S, N, etc.) are influencing the bitumen micro-mechanical behavior.

We simply consider the bitumen as the mixture of asphaltene, naphthene aromatics and saturates. Set the phase-field variable φ_i_ to represent different phase, where φ_1_ is asphaltene, φ_2_ is naphthene aromatics and φ_3_ is saturates.

(1) $$\phi_{1}+\phi_{2}+\phi_{3}=1$$

where the value of φ_i_ (*r*,*t*) represents mass proportion of each phase, and the accurate mass proportion is determined through MD simulation.

Total free energy *F* in a trinary system is given as [31]

(2) $$F=\phi_{1}\ln\phi_{1}+\phi_{2}\ln\phi_{2}+\phi_{3}\ln\phi_{3}+\kappa_{12}\phi_{1}\phi_{2}+\kappa_{13}\phi_{1}\phi_{3}+\kappa_{23}\phi_{2}\phi_{3}$$

where *k_ij_* is the interaction coefficient between phase *i* molecules and *j* molecules, i = 1, 2, 3.

Consider that the system is mass conserved; in that case, the Cahn-Hilliard equation is employed as the governing equation [32,33]

(3) $$\frac{\partial \phi_{i}}{\partial t}+u\cdot \nabla \phi_{i}=\nabla \cdot \left[M\nabla \left(\frac{\partial F}{\partial \phi_{i}}-\nabla \cdot \frac{\partial F}{\partial \left(\nabla \phi_{i}\right)}\right)\right]$$

where u is the velocity, and *M* = 1.2 × 10^–4^ m^2^/s is the mobility parameter that controls the microstructure evolution speed.

The natural boundary condition is used [34,35]

(4) $$\hat{n}\cdot \nabla \phi_{i}=0$$

where $\hat{n}$ is the unit vector to the domain boundary.

Note that we are currently using a simplified three-component asphalt molecular structure and it is very possible that our current simulation could not reflect all the bitumen’s micro-mechanical behaviors. Future studies should focus on a more comprehensive bitumen molecular model.

### 3.2. Microstructure Evolution Direction

Equations (1)–(4) construct the basic phase dynamics system. To fundamentally understand the micro-mechanics in bitumen, the microstructure evolution direction of the free energy in phase dynamics must be evaluated first. In free energy theory, if the bitumen system can overcome one “energy barrier”, the microstructure may develop in one direction; if not, it might develop in the opposite direction. Assume bitumen consists of asphaltene, naphthene aromatics and saturates and consider a binary case (cf. Figure 3), the asphaltene mass fraction is constant, φ = 0 refers to the saturates phase and φ = 1 refers to the naphthene aromatics phase.

Since the bitumen microstructure is a binary phase system (cf. Figure 3), a binary free energy function is used as follows, which is suggested by Lusk et al. [36]

(5) $$f\left(\phi \right)=\frac{1}{4}\eta \phi^{2}{\left(\phi -1\right)}^{2}+\frac{3\epsilon }{2}\left(\frac{\phi^{3}}{3}-\frac{\phi^{2}}{2}\right)$$

where the parameter ε controls the stability. For ε ˃ 0, the stable state is at φ = 1, while for ε ˂ 0, the stable state is at φ = 0. When ε is equal to zero, both φ = 0 and φ = 1 are at metastable state. 

**Figure 5:**
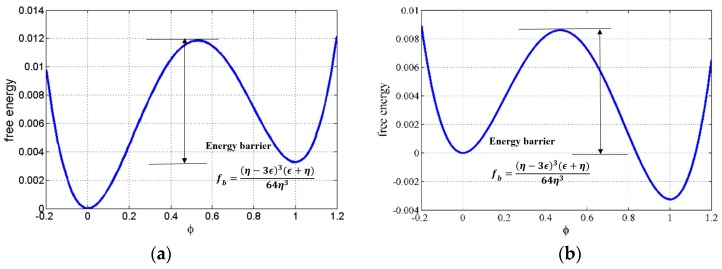
Uneven free energy: (**a**) η = 0.65 and ε = −0.0130; and (**b**) η = 0.65 and ε = 0.0130.

For ε ˂ 0, a typical free energy function (η = 0.65 and ε = −0.0130) is shown in Figure 5. Figure 5a shows an uneven double well potential which means the system does not have two equal minimum states and thus the system does not have the same opportunity of two evolution directions. 

The minimum free energy happens at φ = 0 and this state is stable. There exists a fall between φ = 0 and φ = 1. Thus, the state at φ = 0 is metastable. A small perturbation around the vicinity of φ = 0 may cause the phase to separate to φ = 1. Note that the difference of the two states is $\Delta f=\frac{\epsilon }{4}$, and the energy barrier $f_{b}=\frac{{\left(\eta -3\epsilon \right)}^{3}\left(\epsilon +\eta \right)}{64\eta^{3}}$. Consider two cases: (1) Micro-mixture evolution, where the micro mixture simply means the mixture of different phases at microscale, and the phases can be represented by φ = 1 or φ = 0. Usually, matters with similar chemical potential will diffuse into each other and form one phase. For this case, the physical meaning is that the mixture tends to reach φ = 0 phase other than φ = 1 phase; (2) Microstructure evolution: Define φ = 0 as the cracking state and φ = 1 as the unbroken state. As shown in Figure 5a, if φ = 0 is the lowest energy state, the system will develop to φ = 0, indicating the system will crack; and Figure 5b, if φ = 1 is the lowest energy state, the system tends to develop to φ = 1 state, indicating the system will heal itself. The physical meaning of this energy barrier is that the system has to do such work to overcome the fracture trend for healing in bitumen. For the states around φ = 0, only sufficient large perturbation that helps overcome the energy barrier will lead to the minimum stable state φ = 0. For the states in the vicinity of φ = 1, the system will automatically develop to the minimum state. The evolution process that the system tends to reach the minimum state φ = 1 is actually the process of self-healing.

For ε ˃ 0, a typical free energy function is shown in Figure 5b. It is obvious that the system prefers φ = 1 state, where the minimum value of the system reaches at φ = 1. Similarly, points around the metastable point φ = 0 need a sufficiently large perturbation to reach the stable state φ= 1. Consider two cases. (1) Micro-mixture evolution: For this case, the physical meaning is that the mixture tends to reach the φ = 1 phase other than φ = 0 phase; (2) Microstructure evolution: The physical meaning of this energy barrier is that the system has to do such work to overcome the healing trend other than fracture in bitumen. For both cases, the energy barrier $f_{b}=\frac{{\left(\eta -3\epsilon \right)}^{3}\left(\epsilon +\eta \right)}{64\eta^{3}}$.

The accurate determination of the control parameters η and ε is actually very complex and we plan to evaluate them using Molecular Dynamics Simulation based on bitumen molecular structure and nano-indentation tests on bitumen samples in future studies. It is expected that nano-indentation test can investigate the viscous and adhesion properties of bitumen at microscale. Currently, we use the empirical values based on previous researches [15,16].

### 3.3. Temperature Effects

Another important factor that affects the bitumen microstructure evolution and micromechanical behavior is temperature. The bitumen properties will significantly change from elasticity to viscoelasticity after it reaches the “critical temperature”. The following model suggested by Karma et al. [37] can be used to study the temperature (T) effects on bitumen microstructure evolution

(6) $$f\left(\phi ,T\right)=\frac{\phi^{4}}{4}-\frac{\phi^{2}}{2}+\frac{1}{\omega }\left(\phi -\frac{2\phi^{3}}{3}+\frac{\phi^{5}}{5}\right)T$$

Set the critical temperature as $T_{c}$ and rewrite Equation (6) as

(7) $$f\left(\phi ,T\right)=\frac{1}{2}\phi^{2}{\left(1-\phi \right)}^{2}+\frac{1}{\omega }\left(\phi -\frac{2\phi^{3}}{3}+\frac{\phi^{5}}{5}\right)\left(T-T_{c}\right)$$

**Figure 6:**
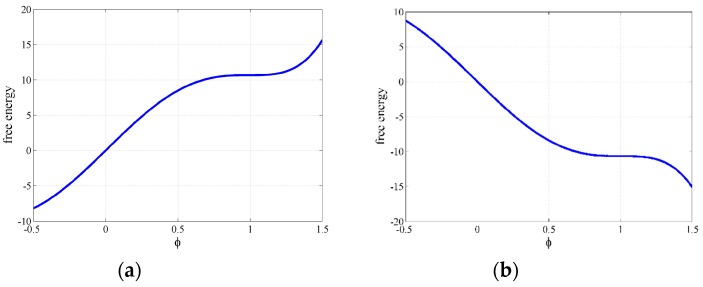
Uneven free energy: (**a**) ω = 1 and *T* − *T_c_* = 20 K; and (**b**) ω = 1 and *T* − *T_c_* = −20 K.

Define φ = 0 as the fluid state of bitumen and φ = 1 as the solid state. The properties of bitumen are a function of temperature. For simplicity, we currently consider that temperature will only affect the microstructure evolution. Consider the case where ω = 1 and *T* − *T*_c_ = 0, the equation decays to the normal double well potential function, which means resin and oil have the same effects on the bitumen microstructure evolution. Considering a low temperature where *T* is lower than *T*_c_, the free energy for ω = 1 and *T* − *T*_c_ = −20 K is shown in Figure 6a. Define φ = 0 as the fluid state of bitumen and φ = 1 as the solid state. One well around φ = 0 has disappeared due to the effect of temperature. The physical meaning is that the bitumen system currently has only one metastable state around φ = 0.5, which indicates that the system prefers solid state of bitumen. For a high temperature, consider the case where ω = 1 and *T* − *T_c_* = 20 K, the free energy is shown in Figure 6a. It is seen that the free energy curve only has one stable state, which indicates that bitumen tends to reach the fluidity state.

### 3.4. Inflection Point Interval

The current phase field model in Equations (1)–(4) is essentially based on the inflection point effects that resin and oil almost have same influences on bitumen microstructure evolution. 

The inflection point is a point on a curve where the curvature or concavity changes sign. For phase field modeling of bitumen microstructure evolution, the state between the inflection points will cause the spinodal decomposition in the system. If the bitumen sample composition is “appropriate”, where the total microstructure free energy lies outside the inflection point interval, the bitumen micro-properties are metastable.

**Figure 7:**
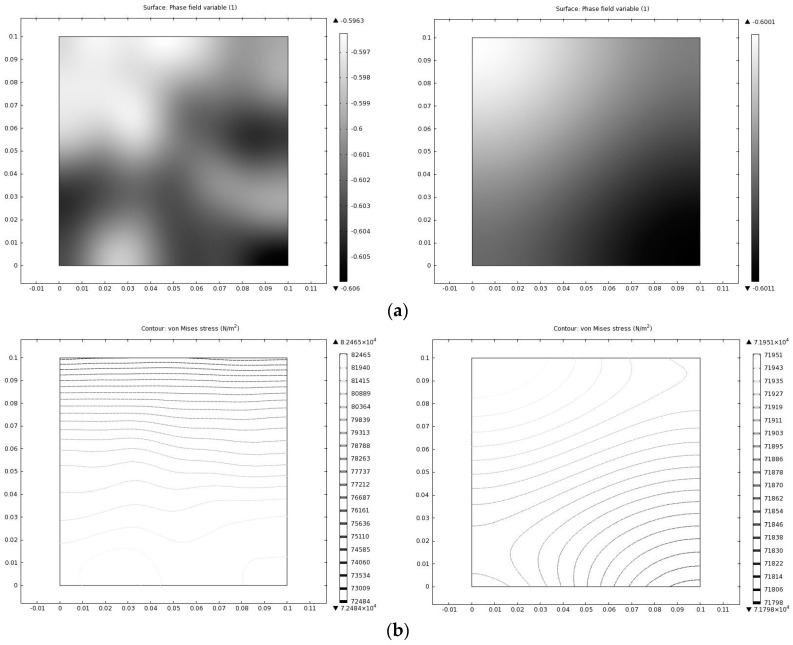
Phase dynamics outside inflection point interval: (**a**) phase distribution at different time instant for states outside the inflection point interval; and (**b**) von mises stress at different time instant for states outside the inflection point interval.

For a free energy function $f={\left(1-\phi^{2}\right)}^{2}$, the system has the minimum energy state at φ = −1 and φ = 1. Set the second derivative of the free energy as zero, and calculate the *x* coordinate of inflection points. A region with *x* coordinate within the inflection point interval will cause spinodal decomposition in the system, while the outside part will not. To test the area outside the inflection points, an initial value φ = −0.6 is set. Figure 7a shows the phase dynamics process in a square region for a state outside the inflection points interval. Since the state is outside the inflection point interval, spinodal decomposition does not happen, which means the system is metastable and no phase separation in the bitumen microstructure system will happen under this condition, indicating the microstructure observed in Figure 3 cannot be seen under such circumstance. The von Mises yield criterion can be used to judge the stress state of the ductile materials including bitumen. Figure 7b shows the von Mises stress of bitumen for a state outside the inflection point interval under certain thermodynamic conditions. It is observed that the stress distributes evenly in bitumen and no significant stress concentration occurs. 

### 3.5. Molecular Structure Construction

**Figure 8:**
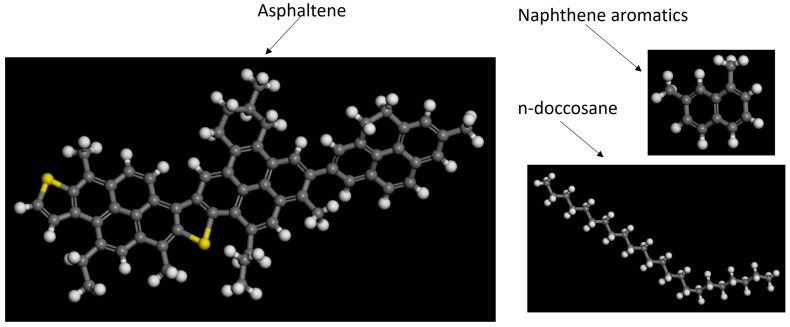
Typical asphaltene/napthene/saturates molecular structures.

Generally, the Phase Dynamics Theory can be used to express the phase in-fusion and separation in bitumen at mesoscale. To more accurately evaluate the process and determine the accurate mass fraction of different phases in bitumen, which is also phase field variable value φ_i_ in the phase field system as shown in Equation (1), the Molecular Dynamics (MD) Simulation is employed to construct the bitumen microstructure. As we mentioned earlier, bitumen is often characterized chemically by its hydrocarbon groups composition, e.g., three main constituent groups including asphaltene, naphthene aromatics and saturates, as shown in Figure 8. Three different types of molecules are used to represent the corresponding constituent species and the three are then formed together to create a bitumen-like ensemble of molecules. This technique was first proposed by Zhang and Greenfield [30]. In their model, 1,7-dimethylnapthalene is used to represent naphthene aromatics and n-C_22_ (n-doccosane) molecules are used to represent saturates. Note that we are currently studying pure bitumen, while the interaction between bitumen and aggregate needs to be considered for a bituminous mixture [38,39].

For the bitumen molecular structure simulation in our study, the COMPASS (Condensed-phase Optimized Molecular Potentials for Atomistic Simulation Studies) force field is used, and it can be described as [40]

(8) $$E_{\mathrm{total}}=E_{b}+E_{\theta }+E_{\phi }+E_{\chi }+E_{b,b\prime }+E_{b,\theta }+E_{b,\phi }+E_{\theta ,\theta \prime }+E_{\theta ,\;\theta \prime ,\phi }+E_{q}+E_{\mathrm{vdW}}$$

where *E*_b_ is the potential energy of bonds; *E*_θ_ is the potential energy of bond angle; *E*_φ_ is the potential energy of dihedral angle; *E*_b,b′_, *E*_b,θ_, *E*_b,φ_, *E*_θ_*,*_θ′_, and *E*_θ,θ′,φ_ are the interaction energy in cross-terms; *E*_q_ is the Coulombic function for electrostatic interactions; and *E*_vdW_ is the potential energy for van der Waals interactions.

**Figure 9:**
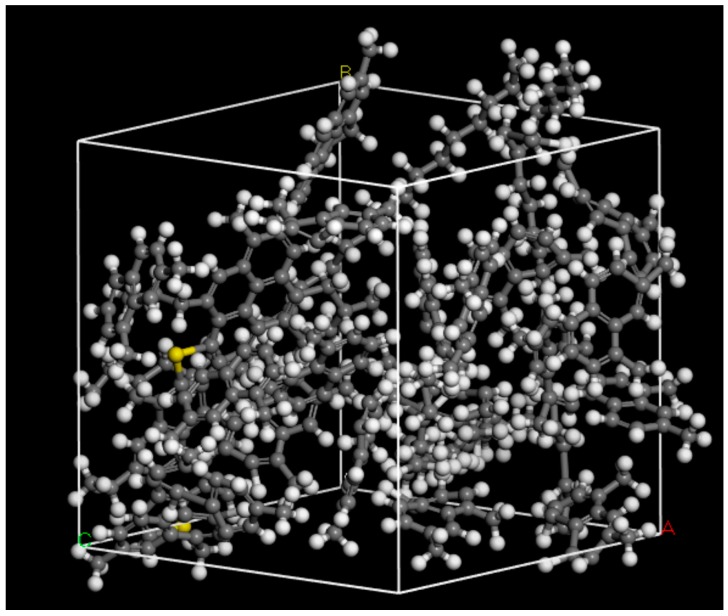
Constructed bitumen molecules ensemble in amorphous cell.

The whole bitumen molecular structure is constructed using Material Studio 7.0 (BIOVIA, San Diego, CA, USA). The amorphous cell is used to construct the representative volume element in bitumen. The target density of the final configurations is set to 1 g/cm^3^ based on Zhang and coworkers’ research [30]. After structure refining and energy minimization, Figure 9 shows one constructed bitumen-like ensemble consisting of 1 asphaltene, 18 1,7-dimethylnapthalene and 3 n-C_22_ (n-doccosane) molecules with mass fraction 19.0:60.2:20.8. The lattice edge length (Å) is 19.8 × 19.8 × 19.8.

To study the effects of resin and oil on bitumen micro-properties, two other mass fraction ratios are selected for construction of bitumen molecular ensemble: (i) 2 asphaltene, 23 1,7-dimethylnapthalene and 12 n-C_22_ (n-doccosane) molecules with mass fraction 19.1:38.8:42.1; and (ii) 3 asphaltene, 69 1,7-dimethylnapthalene and 2 n-C_22_ (n-doccosane) molecules with mass fraction 19.1:38.8:42.1. For each simulation, the periodic boundary conditions are used to eliminate the surface effects. The system temperature is set at 298 K and NVT condition (constant number of atoms, volume and temperature) is used.

The atoms in each molecule in the bitumen-like amorphous cell are then subjected to the conjugate gradient energy minimization within 10,000 fs (1 fs = 1 × 10^–15^ s). The amorphous cell is later refined using molecular dynamics calculation for a time 10.0 ps (1 ps = 1 × 10^–12^ s) with time step 1.0 fs. By applying loading conditions, the stress-strain curve of the bitumen-like ensemble and the elastic constants could be obtained. 

Using the visco-elastic module in Materials Studio software, the modulus is calculated as 1.0539 GPa, 0.9 GPa and 1.4 GPa, respectively. Poisson’s ratio is 0.36. When the values are compared with Figure 3b, it is discovered that first mass proportion, i.e., 19.0:60.2:20.8, of bitumen composition is more close to the actual bitumen, where we finally set φ_1_ = 0.19, φ_2_ = 0.602, and φ_3_ = 0.208 in phase field simulation.

### 3.6. Micro-Mechanical Behavior Evaluation

By considering the microstructure evolution and temperature effects, the free energy Equation (2) is further developed as

(9) $$F=\frac{1}{4}\eta \left(T-T_{c}\right)\left[\phi_{1}\ln\phi_{1}+\phi_{2}\ln\phi_{2}+\phi_{3}\ln\phi_{3}\right]+\frac{3}{2}\epsilon \left(\kappa_{12}\phi_{1}\phi_{2}+\kappa_{13}\phi_{1}\phi_{3}+\kappa_{14}\phi_{1}\phi_{4}+\kappa_{23}\phi_{2}\phi_{3}\right)$$

Substitution of Equation (9) into Equation (3) will result in the governing equation. Initialize the simulation system with a square with length 0.1 m. Eulerian mesh is used for the phase-field computation. Generally, 7–10 mesh points across the interface are required to accurately calculate the diffuse interface. However, if we use a uniform mesh with cell size determined by the interfacial thickness, then the computational mesh will be prohibitively large. To resolve this problem, we use an adaptive mesh which only refines on the interface.

The following physical properties of bitumen are used in the simulation based on our previous researches [15,16]: density ρ = 1010 kg/m^3^, Possion’s ratio υ = 0.4, Young’s modulus *E* = 1.05 MPa, volumetric thermal expansion coefficient α = 6 × 10^–4^, thermal conductivity is 0.75 W/(m·K), and heat capacity is 120 J/(K∙kg).

**Figure 10:**
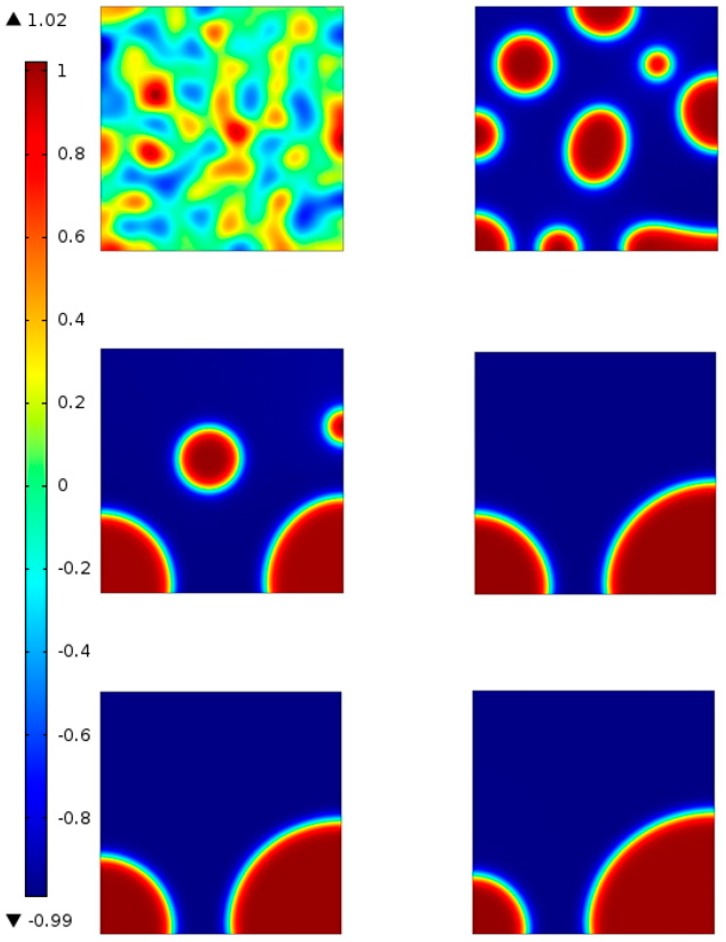
Phase distribution at different time instants under cooling loading.

For cooling the bitumen from *T* = 333.15 K to 273.15 K (60 °C to 0 °C), the phase distribution at different time instant is shown in Figure 10. The snapshots are taken at 0 s, 0.2 s, 0.4 s, 0.6 s, 0.8 s and 1 s. Figure 10 shows that during the decrease in temperature, the original well-mixed phases gradually change into two different phases. There is a clear interface between the two phases.

**Figure 11:**
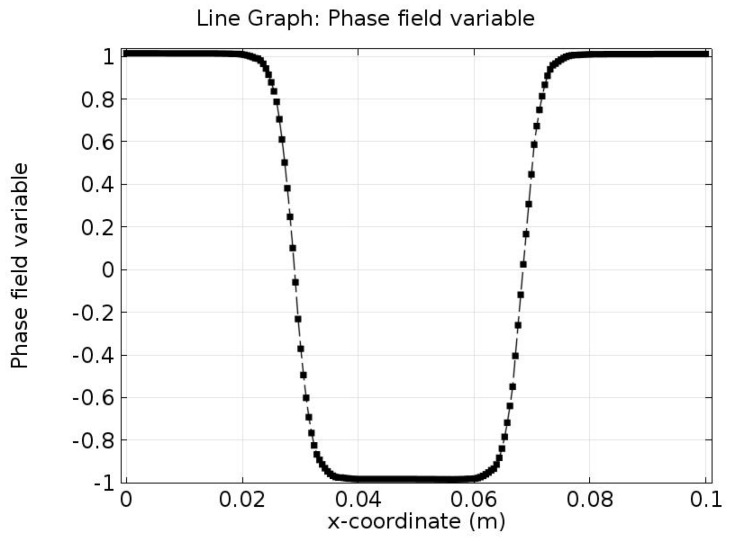
Phase field variable distribution at the bottom boundary at *t* = 0.5 s.

Figure 11 shows the phase field variable distribution on the bottom boundary at *t* = 0.5 s. It is obvious that the diffuse interface exists between the two phases (Phase I and Phase II as shown in Figure 3) represented by 0 and 1.

**Figure 12:**
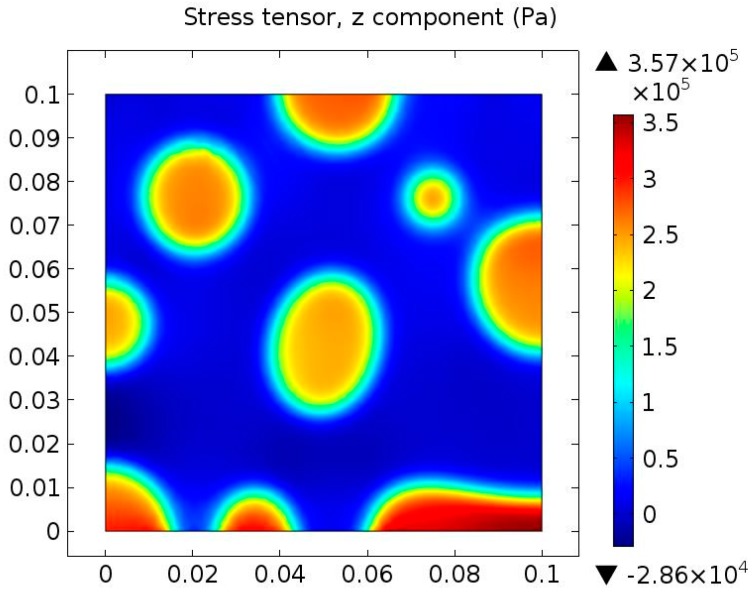
*z* component of stress tensor at *t* = 0.05 s.

The von Mises stress distributes along the interface between saturates and naphthene aromatics. Note that there exists a high von Mises stress zone at the interface. It is easy to conclude that the diffuse interface has a considerably lower stress concentration compared to the sharp interface. The reason why there exists a high stress gradient is because of our 2D assumptions: we assume a “plane-stress” situation since we are studying the bitumen film in AFM experiments and thus in the *z* direction there will exist a high stress zone, which is caused by phase separation as show in Figure 12. The stress concentration area may form discontinuities, e.g., cracks, which further affect the bitumen micro-mechanical behavior.

**Figure 13:**
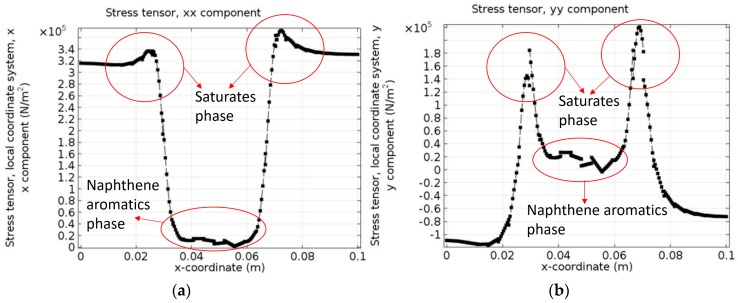
(**a**) *xx* component; and (**b**) *yy* component of stress tensor of bottom boundary at *t* = 0.5 s.

Figure 13a,b presents the *xx* component and *yy* component of the stress tensor of the bottom boundary at *t* = 0.5 s. When compared with the phase separation process at the same time in Figure 10, it is obvious that the middle part of Figure 13a shows very low stress since this region represents the naphthene aromatics. It is expected that the boundary condition and different chemical potentials in different phases contribute to the occurrence of a high stress zone. Besides, due to the existence of the diffuse interface, stress gradually changes across the interface. Figure 13b reveals that the stress σ*_yy_* increases strongly from the naphthene aromatics phase (middle part) to a certain region of the interface (either left or right side). Then, it suddenly decreases to zero and changes of sign after crossing the interface and finally reaches a stable negative value in the saturates phase. The naphthene aromatics phase has almost no stress tensor and the interface is the transition zone from a tensile state to a compressive state. Figure 13a,b indicates that the internal stresses on the bitumen microstructure surface may possibly be caused by saturates phase.

**Figure 14:**
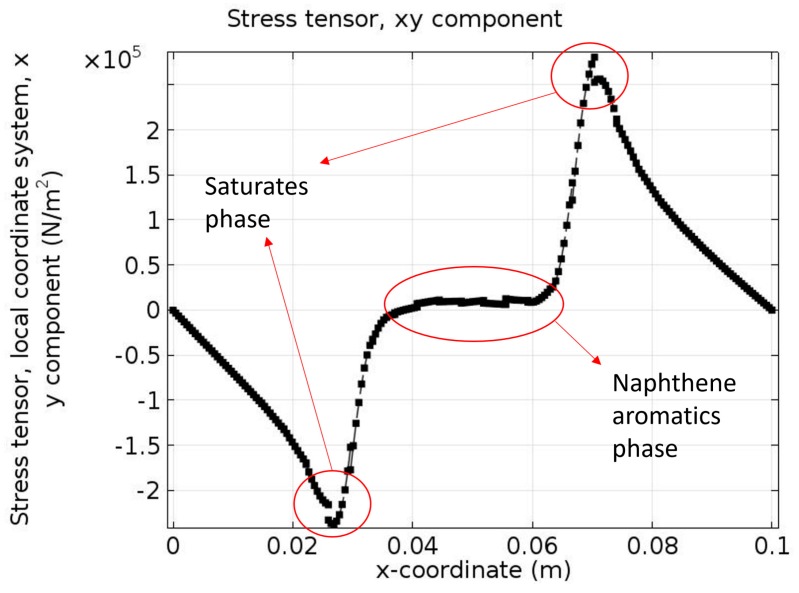
*xy* component of stress tensor of bottom boundary at *t* = 0.5 s.

Figure 14 shows the shear stress tensor σ*_xy_* at the given time instant *t* = 0.5 s. Different signs of the two sides of the plot indicate that shear stresses have different directions. They reach the peak values near the interfaces and in the saturates phase shear stress decreases to zero since two shear stresses induced by the two sides are “neutralized”. The results of Figure 14 further confirm our assumption that the saturates phase plays a major role in the bitumen micro-mechanical behavior.

**Figure 15:**
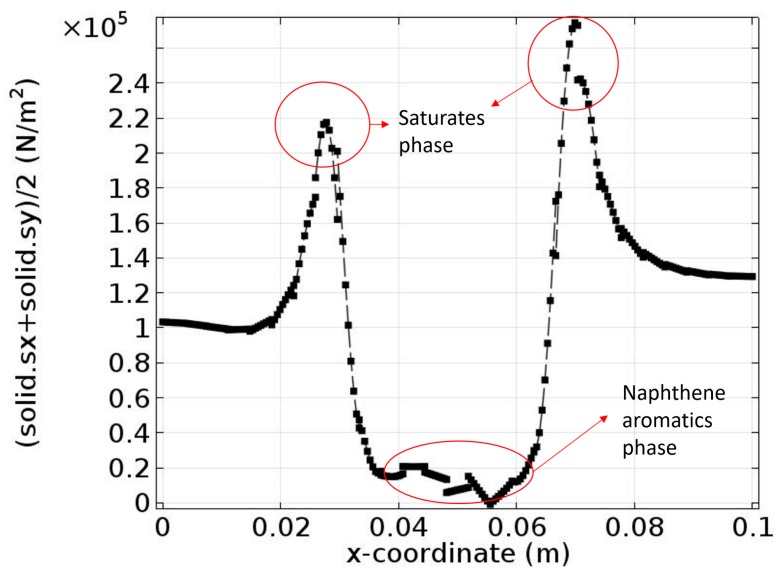
Average stress distribution.

If we combine σ*_xx_* and σ*_yy_* together to get the average stress distribution, we get a result shown in Figure 15, which agrees with the results found by Hu et al. [40]. Calculation results indicate that the peak value of the stress occurs at the interface. The only difference is that we have two peak values while the other has only one peak value. This is because we have two interfaces in the bottom boundary while Hu et al. assume the system has only one interface [41]. 

Based on the above-mentioned analysis, the following conclusions can be obtained:

1. Phase separation does have a significant effect on the bitumen micro-mechanical behavior. Due to the existence of a bi-phase, the stress distribution becomes non-uniform and especially, stresses in some regions of the system show an increase while stresses in some other regions show a large decrease. The stress concentration area may form discontinuities, e.g., cracks, which further affect the bitumen micro-friction behavior.
2. Simulation results also show that the saturates phase, other than the naphthene aromatics phase, plays a major role in the bitumen micro-mechanical process.

## 4. Conclusions

Fundamental understanding of micro-mechanical behaviors in bitumen, including phase separation, micro-friction, micro-abrasion, etc., can help pavement engineers better understand the bitumen mechanical performances at macroscale. Recent researches show that the microstructure evolution in bitumen will directly affect its surface structure and micromechanical properties, which will further affect the micro-mechanical performance. In this study, the bitumen microstructure and micro-mechanical behaviors are studied using Atomic Force Microscopy (AFM) experiments and Phase Dynamics Theory. The AFM experiment results show that different phase-structure will occur at the surface of bitumen samples under certain thermodynamic conditions at microscale. The occurrence of a phase-structure can be explained using phase dynamics theory, where the effects of stability parameter and temperature on bitumen microstructure and micro-mechanical behavior are studied combined with Molecular Dynamics (MD) simulation. Simulation results show that the saturates phase, in contrast to the naphthene aromatics phase, plays a major role in bitumen micro-mechanical behavior. A high stress zone occurs at the interface between saturates phase and the naphthene aromatics phase, which may form discontinuities that further affect the bitumen micro-mechanical performance. Future studies will be focusing on prediction of changes in behavior when the bitumen composition is changed. This could lead to the development of an optimum composition of the bituminous binder based on the groups mentioned in this paper that also shows what happens with the system in the case of polymer modification of the bitumen. 

## Figures and Tables

**Table 1:** Summary on bitumen three-compositions.

Compositions	Features	Average Molecule Weight	C/H Ratio	Content %	Relative Density (Compared with Water)
Oil phase	Transparent liquid	200–700	0.5–0.7	45–60	0.910–0.925
Resin phase	Brown half-solid	800–3000	0.7–0.8	15–30	Lager than 1
Asphaltene phase	Solid	1000–5000	0.8–1.0	5–30	1.100–1.500

## References

[1] Bazlamit S., Reza F. (2005). Changes in Asphalt Pavement Friction Components and Adjustment of Skid Number for Temperature. J. Transp. Eng..

[2] Fischer H., Cernescu A. (2015). Relation of chemical composition to asphalt microstructure—Details and properties of micro-structures in bitumen as seen by thermal and friction force microscopy and by scanning near-field optical microscopy. Fuel.

[3] Al-Rub R., Darabi M., Little D., Masad E. (2010). A micro-damage healing model that improves prediction of fatigue life in asphalt mixes. Int. J. Eng. Sci..

[4] Kanafi M., Kuosmanen A., Pellinen T., Tuononen A. (2015). Macro- and micro-texture evolution of road pavements and correlation with friction. Int. J. Pavement Eng..

[5] Loeber L., Sutton O., Morel J.V.J.M., Valleton J.M., Muller G. (1996). New direct observations of asphalts and asphalt binders by scanning electron microscopy and atomic force microscopy. J. Microsc..

[6] Pauli A.T., Branthaver J.F., Robertson R.E., Grimes W., Eggleston C.M. (2001). Atomic force microscopy investigation of SHRP asphalts: Heavy oil and resid compatibility and stability. Prepr. Am. Chem. Soc. Div. Pet. Chem..

[7] Jäger A., Lackner R., Eisenmenger-Sittner C., Blab R. (2004). Identification of microstructural components of bitumen by means of atomic force microscopy (AFM). PAMM.

[8] Masson J.F., Leblond V., Margeson J. (2006). Bitumen morphologies by phase-detection atomic force microscopy. J. Microsc..

[9] Allen R.G., Little D.N., Bhasin A. (2012). Structural characterization of micromechanical properties in asphalt using atomic force microscopy. J. Mater. Civ. Eng..

[10] McCarron B., Yu X., Tao M., Burnham N. (2011). The Investigation of “Bee-Structures” in Asphalt Binders.

[11] Masson J.-F., Leblond V., Margeson J., Bundalo-perc S. (2007). Low-temperature bitumen stiffness and viscous paraffinic nano and micro-domains by cryogenic AFM and PDM. J. Microsc..

[12] De Mores M.B., Pereira R.B., Simao R.A., Leite L.F.M. (2010). High temperature AFM study of CAP 30/45 Pen grade bitumen. J. Microsc..

[13] Pauli A.T., Grimes R.W., Beemer A.G., Turner T.F., Branthaver J.F. (2011). Morphology of asphalts, asphalt fractions and model wax-doped asphalts studied by atomic force microscopy. Int. J. Pavement Eng..

[14] Dourado E.R., Simao R.A., Leite L.F.M. (2012). Mechanical properties of asphalt binders evaluated by atomic force microscopy. J. Microsc..

[15] Hou Y., Sun W., Das P., Song X., Wang L., Ge Z., Huang Y. (2016). Coupled Navier-Stokes Phase-Field Model to Evaluate the Microscopic Phase Separation in Asphalt Binder under Thermal Loading. J. Mater. Civ. Eng..

[16] Hou Y., Wang L., Pauli T., Sun W. (2015). Investigation of the Asphalt Self-healing Mechanism Using a Phase-Field Model. J. Mater. Civ. Eng..

[17] Yang C., Tartaglino U., Persson B. (2006). A multiscale Molecular Dynamics approach to Contact Mechanics. Eur. Phys. J. E.

[18] Yang C., Persson B. (2007). Molecular dynamics study of contact mechanics: Contact area and interfacial separation from small to full contact. Phys. Rev. Lett..

[19] Campana C., Muser M. (2007). Contact mechanics of real versus randomly rough surfaces: A Green’s function molecular dynamics study. Europhys. Lett..

[20] Fischer H., Stadler H., Erina N. (2013). Quantitative temperature-depending mapping of mechanical properties of bitumen at the nanoscale using the AFM operated with PeakForce Tapping™ mode. J. Microsc..

[21] Yu X., Zaumanis M., dos Santos S., Poulikakos L. (2014). Rheological, microscopic, and chemical characterization of the rejuvenating effect on asphalt binders. Fuel.

[22] Cahn J.W., Hilliard J.E. (1958). Free Energy of a Nonuniform System. I. Interfacial Free Energy. J. Chem. Phys..

[23] Schmets A., Kringos N., Pauli T., Redelius P., Scarpas T. (2010). On the existence of wax-induced phase separation in bitumen. Int. J. Pavement Eng..

[24] Das P., Kringos N., Wallqvist V., Birgisson B. (2013). Micro-mechanical investigation of phase separation in bitumen by combining atomic force microscopy with differential scanning calorimetry results. Road Mater. Pavement Des..

[25] Jahangir R., Little D., Bhasin A. (2014). Evolution of asphalt binder microstructure due to tensile loading determined using AFM and image analysis techniques. Int. J. Pavement Eng..

[26] Koots J.A., Speight J.G. (1975). Relation of petroleum resins to asphaltenes. Fuel.

[27] Lian H., Lin J.R., Yen T.F. (1994). Peptization studies of asphaltene and solubility parameter spectra. Fuel.

[28] Hubbard R.L., Stanfield K.E. (1948). Determination of asphaltenes, oils, and resins in asphalt. Anal. Chem..

[29] (2000). Standard Test Methods of Bitumen and Bituminous Mixtures for Highway Engineering (JTJ 052-2000).

[30] Zhang L., Greenfield M.L. (2007). Analyzing properties of model asphalts using molecular simulation. Energy Fuels.

[31] Hou Y., Wang L., Yue P., Pauli T., Sun W. (2014). Modeling Mode I Cracking Failure in Asphalt Binder by Using Nonconserved Phase-Field Model. J. Mater. Civ. Eng..

[32] Hou Y., Yue P., Wang L., Sun W. (2015). Fracture Failure in Crack interaction of Asphalt Binder by Using a Phase Field Approach. Mater. Struct..

[33] Hou Y., Sun W., Huang Y., Ayatollahi M., Wang L., Zhang J. (2016). Diffuse-Interface Model to Investigate the Asphalt Concrete Cracking Subjected to Shear Loading at a Low Temperature. J. Cold Reg. Eng..

[34] Hou Y., Sun F., Sun W., Guo M., Xing C., Wu J. (2016). Quasi-brittle Fracture Modeling of Preflawed Bitumen Using a Diffuse Interface Model. Adv. Mater. Sci. Eng..

[35] Hou Y., Huang Y., Sun F., Guo M. (2016). Fractal Analysis on Asphalt Mixture Using a Two-Dimensional Imaging Technique. Adv. Mater. Sci. Eng..

[36] Lusk M., Jou H.J. (1997). On the rule of additivity in phase transformation kinetics. Metall. Mater. Trans. A.

[37] Karma A., Rappel W.J. (1998). Quantitative phase-field modeling of dendritic growth in two and three dimensions. Phys. Rev. E.

[38] Guo M., Motamed A., Tan Y.Q., Bhasin A. (2016). Investigating the Interaction between Asphalt Binder and Fresh and Simulated RAP Aggregate. Mater. Des..

[39] Guo M., Tan Y.Q., Zhou S.W. (2014). Multiscale Test Research on Interfacial Adhesion Property of Cold Mix Asphalt. Construct. Build. Mater..

[40] Sun H., Ren P., Fried J. (1998). The COMPASS force field: Parameterization and validation for phosphazenes. Comput. Theor. Polym. Sci..

[41] Hu S.Y., Chen L.Q. (2002). Diffuse-interface modeling of composition evolution in the presence of structural defects. Comput. Mater. Sci..

